# The order of completing MDP and D12 does not affect the breathlessness responses: A randomised controlled trial

**DOI:** 10.1177/14799731251393985

**Published:** 2025-10-30

**Authors:** Isabelle Wemar, Jacob Sandberg, Max Olsson, Josefin Sundh, Magnus Ekström

**Affiliations:** 1 Department of Clinical Sciences Lund, Respiratory Medicine, Allergology and Palliative Medicine, 59568 Lund University Faculty of Medicine, Lund, Sweden; 2 Department of Health, Blekinge Institute of Technology, Karlskrona, Sweden; 3 Department of Respiratory Medicine, Faculty of Medicine and Health, 596174 Örebro University, Örebro, Sweden

**Keywords:** dyspnoea, patient-reported outcomes, dyspnoea-12, multidimensional dyspnoea profile, cardiopulmonary disease, COPD

## Abstract

**Background:**

Breathlessness is a common and distressing symptom across a wide range of medical conditions. Different aspects (dimensions) of breathlessness can be assessed using the Multidimensional Dyspnoea Profile (MDP) and Dyspnoea-12 (D12) questionnaires. We aimed to examine whether the order of completing MDP and D12 affects the breathlessness responses in people with cardiorespiratory disease.

**Methods:**

This was a randomised controlled trial embedded within a longitudinal clinical study. Outpatients with cardiorespiratory disease were randomly assigned to either first complete the MDP or the D12. Primary outcome was mean difference in D12 total score between groups, secondary outcome was mean difference in D12 and MDP subdomain scores. Both outcomes were compared to the minimal clinically important difference (MCID) for each scale.

**Results:**

All 182 participants from the longitudinal study were included. 93 were randomized to complete MDP first and 89 to D12 first. Characteristics such as age, sex, main cause of breathlessness and smoking status were similar between groups. The mean difference for D12 total score (MCID = 2.8) was −1.5 (−4.2 to 1.3, *p* = 0.26) between groups. Mean differences between groups in subdomain scores were also below the corresponding MCID.

**Conclusion:**

The order of completion of MDP and D12 did not impact the scores significantly, but the study lacked power to find smaller yet clinically significant differences. The study supports that the most practical order of completing the instruments can be used in future research and in clinical settings.

## Introduction

Breathlessness is defined as a multidimensional experience of breathing discomfort and is prevalent in people across a wide range of diseases such as chronic obstructive pulmonary disease (COPD) and heart failure.^1^ As breathlessness is associated with poor health outcomes, assessment of breathlessness is important both to evaluate the patient’s symptom burden, changes over time, and the effects of treatment.^2,3^ The gold standard for assessing breathlessness is using patient-reported outcome measures (PROMs). Validated PROMs for assessing different dimensions of breathlessness in people with chronic cardiopulmonary conditions include the Multidimensional Dyspnoea Profile (MDP)^4^ and the Dyspnoea-12 (D12)^5^ questionnaires. D12 was designed to reflect the individuals current everyday experience of breathlessness and intended primarily for clinical settings.^5^ MDP was designed to assess breathlessness in relation to specific events and intended for both clinical and laboratory use.^4^ Despite similarities in clinimetric properties, the instruments may not be substituted for each other and may provide complementary information when used concurrently.^6^ However, when several PROMs are administered consecutively, the sequence of administration may affect the symptom responses – the instructions and ratings of earlier questionnaires may introduce systematic differences (bias) in responses to subsequent questionnaire by priming the mindset of the individual, causing an “order effect”.^7–10^ Despite the importance of breathlessness as a symptom, it has not been investigated whether the order of completing the MDP and D12 affects the symptom responses. Both tools include several emotional aspects which could lead to unintentional priming of the tool coming second. It is hypothesised that if this sort of priming is in place, the effect would be stronger when completing D12 second to MDP, as MDP is more complicated and therefore more time consuming.

The primary aim of this study was to evaluate whether the order of completing MDP and D12 affects the rating of the D12 total score among people with cardiopulmonary disease. Secondary aims were to evaluate order effects on the MDP and D12 subdomain scores and whether any differences were statistically as well as clinically significant.

## Methods

### Design and population

This randomised controlled trial (RCT) was embedded in a longitudinal cohort study with the primary aim of clinically validating the MDP and D12 in Swedish. Patients from internal medicine outpatient clinics and primary care facilities in Karlskrona, Umeå, Uppsala, Stockholm and Örebro were recruited during routine appointments during the years 2016-2017.^11,12^

**Figure 1.**
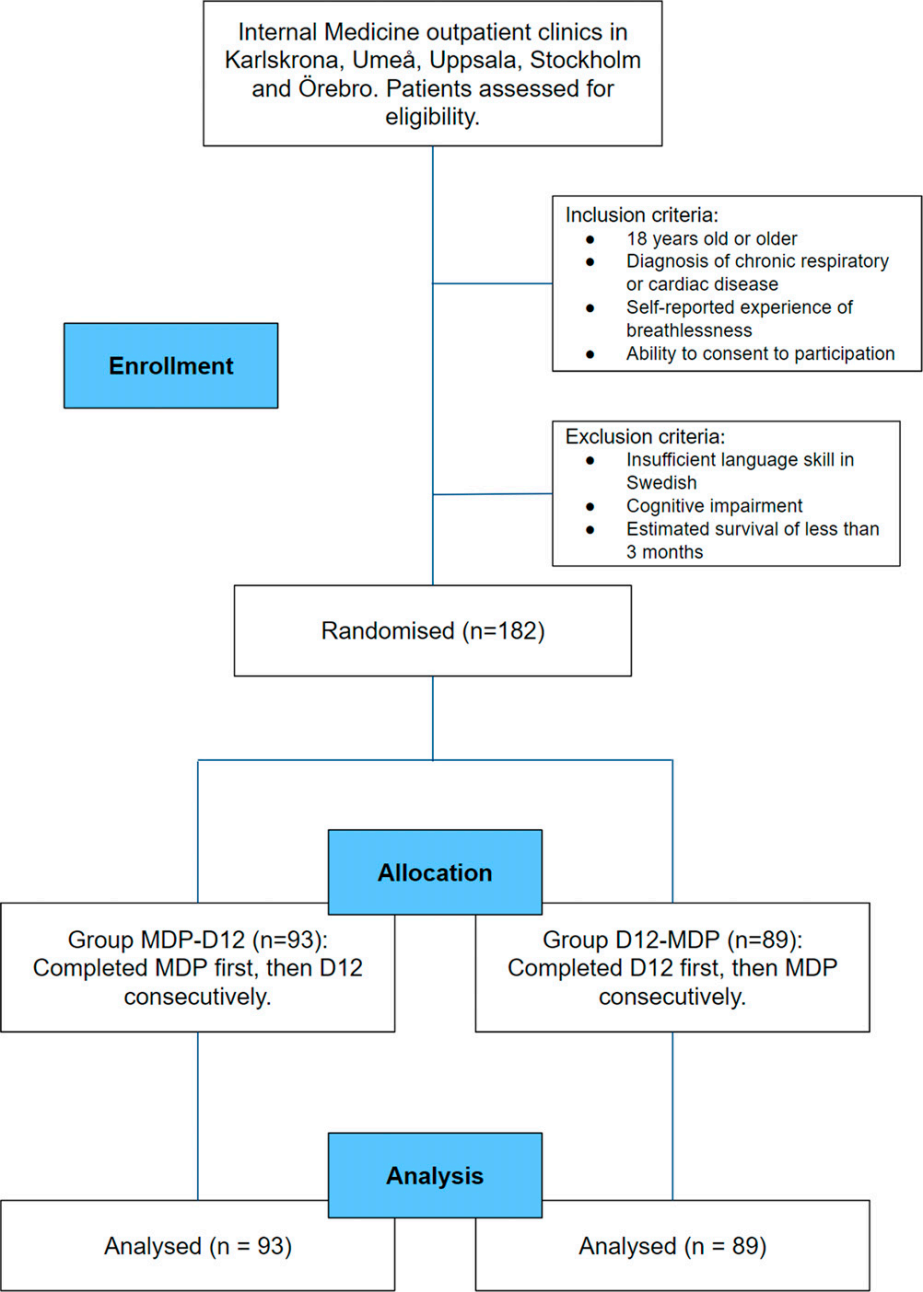
Participant flow chart. *Abbreviations*. *D12,* Dyspnoea-12; *MDP,* Multidimensional Dyspnoea Profile.

MDP and D12 were distributed in a random order to participants using a parallel trial design (Figure 1). Results and procedures were recorded and presented according to CONSORT guidelines.^13^ No amendments to the protocol were made after trial commencement.

Inclusion criteria for the longitudinal study were: age 18 years or older; documented physician-diagnosed chronic respiratory and/or cardiac disease; self-reported breathlessness during daily life defined as an affirmative answer to the question “Did you experience any breathlessness during the last 2 weeks?”; and ability to provide written informed consent to participate in the study. The focal period of 2 weeks in the screening question was chosen to reflect clinical breathlessness in everyday life and enable data comparison with other studies in the field.^6^

Exclusion criteria for the longitudinal study were: inability to write or understand Swedish adequately to participate; cognitive or other inability to participate in the study; and estimated survival less than 3 months.

No participants included in the observational study were excluded from this RCT.

### Randomisation and interventions

Linguistically validated Swedish versions of MDP^14^ and D12^15^ were given to the participants, with the order of MDP and D12 randomised beforehand and then distributed using a sealed opaque envelope opened by the responsible researcher at each participating center at the time of inclusion. The recall period used for MDP and D12 was “during the recent 2 weeks”. The group receiving MDP first, then the D12, was called MDP-D12, and the group receiving D12 first, then MDP was called D12-MDP.

### Assessments and outcomes

At the baseline visit, sex, diagnosed disease, body mass index (BMI) and main cause of breathlessness were obtained from medical records, and smoking status from patient questionnaires.^11^ All outcome measurements were obtained from the first completion of MDP and D12 at baseline visit. For all outcome scores, higher values reflect more severe breathlessness symptoms. D12 consists of 12 questions spanning 1 page; 7 which pertain to physical aspects and 5 which pertain to affective aspects, creating two subdomains (i.e. D12-physical and D12-affective).^5^ MDP consists of 3 subdomains spanning 4 pages; sensory qualities (SQ), breathing discomfort (A1) and emotional responses (A2). SQ and A1 can be combined into a larger subdomain called immediate perceptions (IP).^4^ To assess clinical significance of the results, all outcomes were evaluated against the published minimal clinically important difference (MCID) for the scale.^16^ The primary outcome was the D12 total score (range 0-36, MCID 2.8). Secondary outcome was the subdomain scores for D12 and MDP; D12-physical (range 0-21, MCID 1.8); D12-affective (range 0-15, MCID 1.1); MDP A1 score (range 0-10, MCID 0.8); MDP IP (range 0-60, MCID 4.6); and MDP A2 (range 0-50, MCID 2.4).

### Sample size

The sample size was determined by the primary longitudinal study, allowing for validation of the MDP and D12 questionnaires. For the analysis of this embedded RCT, a power calculation was performed based on the primary outcome (D12 total score) using the actual sample size of the MDP-D12 group (*n* = 93) and the D12-MDP group (*n* = 89), an alpha of 0.05 and aimed at detection of MCID for D12-total of 2.8 units, with a standard deviation of 9 units. Based on these data, the actual power of the present RCT to detect MCID in the primary outcome was 58%. For a power of 80%, a mean difference in D12 total score of 3.8 units (1.4 MCID) could be detected, corresponding to a small to moderate effect.^16^

### Statistical analyses

A statistical analysis plan was completed before the start of analysis. Characteristics were compared between the randomised groups using standard descriptive statistics. Responses for MDP and D12 were then grouped according to the questionnaire order and compared using linear regression. The mean difference was calculated with a 95% confidence interval (CI). Statistical significance was defined as a *p*-value <0.05 for the primary outcome. Clinical significance was defined as a mean between-group difference above the MCID for each scale. Statistical analyses were conducted using the software packages Stata, version 18 (StataCorp LP; College Station, TX).

### Ethical considerations

This RCT was not registered because it was a secondary analysis embedded in a primary longitudinal study. The primary study was registered and approved by the Regional Ethical Board at Lund University (DNr: 2016/16).^11,12^ Participants were informed on the overall purpose of the longitudinal study (to evaluate the D12 and MDP questionnaires) but were not informed about the presence or specific aims of this RCT. Permission for the study was given from the copyright holders for D12 (Professor Janelle Yorke, UK) and MDP (Professor Robert B. Banzett, USA).

## Results

We included a total of 182 participants (Table 1) and 93 of these were randomised to completing MDP first (MDP-D12 group) and 89 to completing D12 first (D12-MDP group). Participant characteristics were the following: in total 53% were female, the mean age was 67 and most were current or former smokers (58%). The most common main underlying condition was COPD (25%) but other causes included asthma (21%), heart failure (19%) and idiopathic pulmonary fibrosis (19%). In the primary outcome, the between-group mean difference in the D12-total score was −1.5, 95% CI -4.2 to 1.3, *p*-value = 0.26; less than MCID = 2.8. For the secondary outcome, i.e., MDP and D12 subdomain scores, the mean differences between groups were also smaller than MCID: D12-physical −0.56, 95% CI -2.1 to 1.0, MCID = 1.8; D12-affective −1.2, 95% CI -2.5 to 0.12, MCID = 1.1; MDP A1 0.1, 95% CI -0.7 to 0.8, MCID = 0.8; MDP IP -2.2, 95% CI -6.2 to 1.8, MCID = 4.6; MDP A2 -0.8, 95% CI -5.4 to 3.8, MCID = 2.4 (Table 2). None of the between-group mean differences for both primary and secondary outcomes were statistically significant as all 95% CIs included zero.

**Table 1.** Baseline characteristics of study participants.

Characteristic		Randomised group	
		MDP-D12 (*n* = 93)	D12-MDP (*n* = 89)
Age (years)	Mean (SD)	66 (14)	68 (13)
Sex, *n* (%)	Female	51 (55)	46 (52)
Main cause of breathlessness, *n* (%)	Chronic obstructive pulmonary disease	22 (24)	23 (26)
	Asthma	21 (23)	18 (20)
	Heart failure	20 (22)	15 (17)
	Idiopathic pulmonary fibrosis	17 (19)	17 (19)
	Other interstitial lung disease	5 (5)	5 (6)
	Other	6 (7)	10 (11)
Smoking status, *n* (%)	Never	6 (7)	7 (8)
	Sometimes	4 (4)	2 (2)
	Formerly daily	57 (63)	50 (56)
	Daily	24 (26)	30 (30)
BMI (kg/m^2^), *n* (%)	Underweight (<19.9)	1 (1)	4 (5)
	Normal (20-24.9)	27 (29)	36 (41)
	Overweight (25-29.9)	32 (34)	26 (30)
	Obesity (≥30)	33 (35)	22 (25)

“MDP-D12” denotes the group who completed MDP before D12 and “D12-MDP” denotes the group who completed D12 before MDP. *Abbreviations*. *BMI* body mass index, *D12* Dyspnoea-12, *MDP* Multidimensional Dyspnoea Profile, *SD* standard deviation.

**Table 2.** Mean scores on the questionnaires and mean differences between groups.

	MDP-D12 group (*n* = 93)	D12-MDP group (*n* = 89)	Mean difference between the groups (95%CI)	MCID for the scale
D12-total	14.1 (9.3)	15.6 (8.8)	−1.5 (−4.2 to 1.3)	2.8
D12- physical	9.5 (5.3)	10.1 (5.2)	−0.56 (−2.1 to 1.0)	1.8
D12-affective	4.5 (4.4)	5.7 (4.4)	−1.2 (−2.5 to 0.12)	1.1
MDP A1	5.0 (2.4)	4.9 (2.7)	0.1 (−0.7 to 0.8)	0.8
MDP IP	23.9 (15.1)	24.7 (14.7)	−2.2 (−6.2 to 1.8)	4.6
MDP A2	14.9 (13.6)	17.1 (13.4)	−0.8 (−5.4 to 3.8)	2.4

Standard deviation values are shown within parentheses in columns “MDP-D12” and “D12-MDP”. “MDP-D12” denotes the group who completed MDP before D12 and “D12-MDP” denotes the group who completed D12 before MDP. MCID values obtained from a study by Ekström et al..^16^ *Abbreviations*. *CI* confidence interval, *D12* Dyspnoea-12, *MCID* minimal clinically important difference, *MDP* Multidimensional Dyspnoea Profile.

## Discussion

### Main findings

The main finding of this multicenter RCT is that the order of completing D12 and MDP did not seem to impact responses on these questionnaires statistically nor clinically, supporting that the instruments can be distributed in the most practical order in future research and clinical settings.

### What this study adds

This is the first study to indicate that randomisation of completion order may not be a necessary measure when using both MDP and D12 in a research setting. Use of both questionnaires can contribute concomitant information on breathlessness experience, which can be useful in both research and clinical settings, but completing multiple assessments consecutively can be a burden on participants.

Both the D12 and the MDP only comprise items with negative and unwanted statements in relation to breathlessness, such as “unpleasant”, “distressing” “chest tightness” or “air hunger”, which could be a reason why we did not find any order effect. A negative statement could potentially have more influence on a subsequent positive statement related to breathing, such as “pleasant” or “calm”.

Measured outcome can be influenced by the visual design of questionnaires, especially the display of items on separate pages [8]. Although MDP consists of four pages and D12 of only one page, the difference in visual design did not seem to introduce an order effect.

### Strengths and limitations

Strengths of this study include the RCT design, the contribution of further evaluation of validated instruments and the analysis of both statistical and clinical differences. The characteristics of randomised groups were comparable, which supports the validity of the randomisation. The participants had cardiorespiratory diagnoses relevant to the questionnaires and other breathlessness research.

Limitations to the study include the sample size being determined by the primary longitudinal study, only allowing for a power of 58% to detect MCID (2.8 units) in the primary outcome. No differences larger than MCID were found in the primary and secondary outcomes, but only a difference in D12 total score of 1.4 MCID (3.8 units) could be detected with 80% power. Hence, the study lacked power to rule out smaller, yet clinically relevant, differences. Additionally, there were too few participants to evaluate outcomes relating to specific cardiorespiratory diagnoses. In this study, only data from the first assessment (baseline visit) of the main longitudinal study was used, although participants completed both instruments at later assessment points (after 2 weeks and 6 months). The present findings apply mainly to chronic breathlessness, as the participants were recruited from outpatient clinics and had cardiorespiratory diagnoses and responded affirmatively to the question “Did you experience any breathlessness during the last 2 weeks?”. The recall period for D12 and MDP was set to the recent 2 weeks to represent everyday breathlessness experiences and allow for comparison with similar validation studies, which is more in line with the original time frame of D12 than the one of MDP, which is flexible and can be set by the user. This could potentially have some negative impact on the accuracy of the MDP scores.

### Implications

Findings from this study can be used both when designing future larger studies in the area of breathlessness research, as well as in the clinical setting when exploring patients’ levels of breathlessness. The most practical order of the instruments can be used in upcoming studies, depending on what degree of difference (i.e., clinical or statistical) needing to be detected.

This study used PROMs printed onto paper, but online surveys are becoming more common in both research and clinical settings. Further studies should evaluate order effects from the D12 and MDP in online surveys, as D12 and MDP differ in grid design, which has been shown to affect questionnaire outcomes when completed on computers or smart phones.^17^

## Conclusion

This RCT aimed at investigating whether the order of consecutive completion of MDP and D12 had significant impact on their scores. No signs of bias or priming could be found but power was too low to rule out MCID. The study supports that the most practical order of completing the instruments can be used in future research and in clinical settings.
